# Vaping Exacerbates Coronavirus-Related Pulmonary Infection in a Murine Model

**DOI:** 10.3389/fphys.2021.634839

**Published:** 2021-05-10

**Authors:** Vijay Sivaraman, De’Jana Parker, Rui Zhang, Myles M. Jones, Rob U. Onyenwoke

**Affiliations:** ^1^Department of Biological and Biomedical Sciences, North Carolina Central University, Durham, NC, United States; ^2^Department of Respiratory and Critical Care Medicine, General Hospital of Ningxia Medical University, Yinchuan, China; ^3^Biomanufacturing Research Institute and Technology Enterprise, North Carolina Central University, Durham, NC, United States; ^4^Department of Pharmaceutical Sciences, North Carolina Central University, Durham, NC, United States

**Keywords:** coronavirus, inflammation, E-cigarette, spirometry, cytokines

## Abstract

Though the current preponderance of evidence indicates the toxicity associated with the smoking of tobacco products through conventional means, less is known about the role of “vaping” in respiratory disease. “Vaping” is described as the use of electronic cigarettes (E-Cigarettes or E-Cigs), which has only more recently been available to the public (∼10 years) but has quickly emerged as a popular means of tobacco consumption worldwide. The World Health Organization (WHO) declared the SARS-CoV-2 outbreak as a global pandemic in March 2020. SARS-CoV-2 can easily be transmitted between people in close proximity through direct contact or respiratory droplets to develop coronavirus infectious disease 2019 (COVID-19). Symptoms of COVID-19 range from a mild flu-like illness with high fever to severe respiratory distress syndrome and death. The risk factors for increased disease severity remain unclear. Herein, we utilize a murine-tropic coronavirus (beta coronavirus) MHV-A59 along with a mouse model and measures of pathology (lung weight/dry ratios and histopathology) and inflammation (ELISAs and cytokine array panels) to examine whether vaping may exacerbate the pulmonary disease severity of coronavirus disease. While vaping alone did result in some noted pathology, mice exposed with intranasal vaped e-liquid suffered more severe mortality due to pulmonary inflammation than controls when exposed to coronavirus infection. Our data suggest a role for vaping in increased coronavirus pulmonary disease in a mouse model. Furthermore, our data indicate that disease exacerbation may involve calcium (Ca^2+^) dysregulation, identifying a potential therapeutic intervention.

## Introduction

From late December 2019 to current, the novel coronavirus SARS-CoV-2 has emerged as a highly communicable respiratory virus. While many patients infected with SARS-CoV-2 do not exhibit severe or life-threatening symptoms, approximately 5% go on to develop the potentially lethal disease known as COVID-19 (Bray et al., 2020). COVID-19 has a significant impact on the pulmonary system that includes pneumonia-like bilateral infiltrates, which are clearly visible by X-ray (Omer et al., 2020). Many patients go on to require oxygen and often need to be mechanically ventilated. In addition to COVID-19 lung disease, there are additional extrapulmonary effects, which include cardiomyopathy and potentially neurological and renal effects. To date, there are no effective therapies for COVID-19, and the mortality rate remains high, both nationally and worldwide. While disease burden appears to be more significant among older minority populations, the disease is significant worldwide regardless of age, gender, or racial associations (Bray et al., 2020). It is likely that unknown risk factors may contribute to disease severity and must, therefore, be further investigated (Tsatsakis et al., 2020).

Electronic cigarettes (E-Cigs) are a relatively new and novel method of tobacco consumption. E-Cigs differ from conventional cigarettes in that they contain no combustible tobacco but rather utilize a battery-operated coil to heat and aerosolize the nicotine (or marijuana or CBD if present) in a liquid vehicle (e-liquid) to the lungs (Besaratinia and Tommasi, 2014). In a relatively short time, E-Cig sales and usage have penetrated most countries worldwide, with high levels of usage in Asian, European, and American markets (Hammond et al., 2019; Huang et al., 2019; Mallock et al., 2020). This relatively new and fast-growing subset of nicotine users, described as “vapers” rather than smokers, utilize products that are very efficient at delivering nicotine so that plasma nicotine levels comparable to those observed with conventional tobacco smoking have now been recorded (Etter and Bullen, 2011). However, since E-Cigs have only recently been available to the population at large (∼10 years), relatively little is known about their physicochemical properties and as to whether long-term E-Cig use will result in respiratory diseases similar to cigarette smoke, none at all, or something entirely different (Davis et al., 2017; Urman et al., 2018). Initially generally regarded as safe (GRAS) by the FDA, recently accumulated data indicate the adverse effects of E-Cig intake and have demonstrated the need for better assessment and regulation of e-liquids as necessary for population safety (Orzabal et al., 2019). Furthermore, there is concern that E-Cig consumption may emerge as an additional variable contributing to increased severity of COVID-19-related pulmonary disease across the worldwide population (Tsatsakis et al., 2020).

Published work has demonstrated murine hepatitis virus (MHV) as a causative agent of SARS-like pneumonia after intranasal exposure in mice (Yang et al., 2014). As is the case with both SARS-CoV1 and CoV2, MHV is a class II beta-coronavirus that also uses a spike protein to enter cells. However, whereas SARS-CoV-2 binds to the angiotensin-converting enzyme 2 (ACE2) and is then cleaved by ACE2 and TMPRSS2 during the entry process (which may also involve cleavage by the intracellular convertase furin), MHV spike protein binds to carcinoembryonic antigen-related cell adhesion molecule 1 (CEACAM1) (Hingley et al., 1998; De Albuquerque et al., 2006; Shang et al., 2020). Although MHV-A59 receptor binding differs from SARS-CoV-2, MHV-A59 spike protein is also cleaved by furin and trypsin-like serine proteases. When administered to mice intranasally (IN) at sub-lethal doses, MHV-A59 causes progressive pneumonia that is characterized by alveolar damage, severe weight loss, and inflammation, including significant increases in IL-1β, IL-6, and TNFα (Yang et al., 2014). A similar lung disease has been observed with the MHV-1 strain (De Albuquerque et al., 2006). Together, these data indicate that MHVs are excellent models of SARS/MERS-like disease. We have recently developed a novel method to expose mouse models and cell lines to an intranasal (IN) vaped e-liquid condensate, which we will herein refer to as “vape” or a “vaped” e-liquid, and used this reagent to evaluate *in vivo* pulmonary function and *in vitro* toxicity. Using this model, we questioned how “vaped” mice would respond to a coronavirus pulmonary challenge.

## Materials and Methods

### Materials

Unless otherwise noted, all reagents and materials were purchased from either Thermo Fisher Scientific (Waltham, MA, United States) or Sigma-Aldrich (St. Louis, MO, United States) at the highest level of purity possible. A 50-mM 2-APB stock was first made up in DMSO, which was then diluted with sterile PBS to 500 μM, as has been previously described for the intravenous use of 2-APB in a mouse model (Morihara et al., 2017).

### Purchase of E-Liquid Products and Vape Distillate Generation

The e-liquids (“Mint” JUUL pods) were purchased locally from retailers in Durham, NC, United States, between July 1, 2020 and October 15, 2020. These products were inventoried and stored at room temperature until used. Manufacturer’s label information stated that ingredients include only vegetable glycerin (VG), propylene glycol (PG), nicotine, flavoring, and benzoic acid, with each pod containing 0.7 ml of the flavored fluid at 3% nicotine.

The e-liquid was vaped using a previously described (Panitz et al., 2015) apparatus to produce an e-liquid vapor distillate. Briefly, e-liquid vapors were produced using a JUUL E-Cig device (battery powered with a prefilled pod) connected to a silicon tubing and to the mouthpiece of the JUUL E-Cig on one end. The other end was placed in the lower part of a 50-ml conical tube, in which the distillate was condensed and collected, suspended above liquid nitrogen inside a thermal container. The JUUL device was utilized for periods of up to 5 s with at least 10 s between activations to simulate “puffs.” To reduce the chance of “dry puffing” the e-liquid pods, only three-fourths of a pod fluid was vaped, which occurred over an ∼3-h duration per pod. The vaped e-liquid condensates were then stored at −20°C until they were used.

The vaped e-liquid was analyzed for nicotine content using a previously published (Pagano et al., 2015) GC/MS protocol (Mass Spectrometry Facility, Louisiana State University, Baton Rouge, LA, United States) and determined to contain a nicotine concentration of ∼2 mg/ml, which is in contrast to the unvaped e-liquid, 35 mg/ml (Jackler and Ramamurthi, 2019). Because each JUUL pod (0.7 ml) can produce ∼200 “puffs”^1^, a “puff” should contain ∼125 μg of nicotine. However, for our studies, we utilized 10-μl (20 μg nicotine) doses of our vaped e-liquid (∼2 mg/ml) in an attempt to minimize any potential morbidity after assessing the effects of several volumes (5, 10, and 20 μl). During gross examination, the latter two doses did not exhibit a noticeable difference (data not shown). Therefore, and again being mindful of minimizing potential morbidity, we chose to use the 10-μl dose.

### Cells and Virus

A549 (CCL-185) and CALU-3 (HTB-55) cells were also obtained from ATCC and maintained in DMEM (Dulbecco’s modified Eagle medium) and MEM (minimum essential medium) alpha (Gibco, Thermo Fisher Scientific), respectively, supplemented with 10% fetal bovine serum (GE Healthcare-HyClone, VWR International) and 100 U/ml of penicillin and 100 μg/ml of streptomycin (BioWhittaker-Lonza from VWR International), 1% L-glutamine (GE Healthcare-HyClone), 1% non-essential amino acids (GE Healthcare-HyClone), and 1% pyruvate (Gibco). Both cell lines were routinely cultured per their instructions and maintained at 37°C in a humidified atmosphere of 5% (vol/vol) CO_2_.

Coronavirus strain MHV-A59 and delayed brain tumor (DBT) cells were kind gifts from the laboratory of Ralph Baric (The University of North Carolina at Chapel Hill, Chapel Hill, NC, United States). DBT cells were maintained in Eagle’s minimum essential medium supplemented with 10% fetal bovine serum, 0.05 μg/ml of gentamicin and 0.25 μg/ml of kanamycin. DBT cells express a relatively uniform and abundant amount of MHVR, the receptor for MHV-A59 docking and entry into cells. Thus, the virus was both generated and quantified [by standard plaque assay to determine plaque-forming units (PFUs), which are also known as infectious units (IUs) (Langlet et al., 2007)] using DBT cells for all experiments.

### Cell Treatments and Viability Assay

A549 (2.0 × 10^4^) or CALU-3 (3.0 × 10^4^) cells were plated in 96-well black-walled tissue culture dishes (Costar catalog #3603 Millipore-Sigma; St. Louis, MO, United States) and grown overnight. E-liquids were serially diluted into the appropriate complete medium to produce the desired final concentrations and administered to the cells for 24 h.

Cell viability was determined using a resazurin (7-hydroxy-3H-phenoxazin-3-one 10-oxide)-based assay (Acros Organics; Fair Lawn, NJ, United States). The resazurin stock solution (1 mg/ml) was prepared in diH_2_O and added to the 96-well assay plates for a final concentration of 0.1 mg/ml. After the treatments, 10 μl of the dye was added to 100 μl of complete culture medium in each well. After 3 h of incubation in 5% CO_2_ (37°C), fluorescence was measured using a PHERAstar Microplate Reader (BMG Labtech; Durham, NC, United States) and the appropriate filter set (ex: 540 nm, em: 590 nm). The relative fluorescence of the mock-treated cells was then arbitrarily converted to 100% for cell viability.

### Mice and Treatments

All mice were obtained from The Jackson Laboratory (Bar Harbor, ME, United States). Young adult mice (6- to 8-week-old male and female C57-BL/6J) were used for all experiments (Finlay and Darlington, 1995). After being received, the mice were allowed to acclimate and recover from shipping stress for 1 week in the NCCU Animal Resource Complex, which is accredited by the American Association for Accreditation of Laboratory Animal Care. All animal care and use were conducted in accordance with the guide for the care and use of the laboratory animals (National Institutes of Health), and mice were maintained at 25°C and 15% relative humidity with alternating 12-h light/dark periods.

Once acclimated, mice were provided anesthesia (isoflurane via a SomnoSuite system), and the e-liquid distillate, vehicle control [50:50 (vol/vol) PG/VG] or saline (10 μl) was delivered dropwise intranasally (IN) using a micropipette, as has been previously described (Miyashita et al., 2018; Gotts et al., 2019), once daily for 3 days to each animal in the appropriate treatment group. After these initial treatments, MHV-A59 infection proceeded IN within a 24-h time period, with the mice anesthetized via an i.p. injection of ketamine (100 mg/kg) and xylazine (50 mg/kg) prior to infection.

Previous studies have demonstrated peak effects of IN infections with 1.5 × 10^4^–1.5 × 10^6^ PFU MHV-A59/mouse at days 5–6 post-infection (p.i.). For our studies, the mice were inoculated with MHV-A59 or vehicle (naïve), and body weight was monitored daily per IACUC protocols for 2–8 days p.i. We chose a broad time period to capture potential differences in infection outcomes between treatments (Figure 2A). At time points of experimental completion, mice were humanely euthanized using an overdose of sodium pentobarbital, as per our accepted animal protocol.

### Cytokine Analysis

At experimental endpoints, bronchoalveolar lavage (BAL) was performed, and supernatants were isolated for cytokine analysis. Inflammatory cytokine proteins were evaluated using ELISA (OptEIA, BD Pharmingen) or Millipore Milliplex reagents and a Luminex 200 system (Millipore Sigma, Burlington, MA, United States).

### Spirometry

Spirometry analysis was conducted using a SomnoSuite low-flow anesthesia system (Kent Scientific Corporation, Torrington, CT, United States). Briefly, mice were sedated with ketamine/xylazine and then attached to a nose cone to monitor average peak CO_2_ wave forms for ∼2–3 min.

### Histopathology

At time points of experimental completion, mice were injected with an overdose of sodium pentobarbital, and then lungs were inflated with 1 ml of 10% neutral-buffered formalin, then removed and suspended in 10% formalin for 12 h. Lungs were washed once in PBS and then immersed in 70% ethanol. Tissues were then embedded in paraffin, and three 5-μm sections 200 μm apart per lung were stained with hematoxylin/eosin (H&E) for examination by the NCCU histopathology core (directed by Dr. X. Chen). Sections were evaluated blindly for gross pathology, and disease score was evaluated as a measure of average pixel number density (pixelation) from multiple images per group using ImageJ software (Harris et al., 2018).

### Statistics

Power analysis was performed using α = 0.05 and power at 0.7. Statistics for analysis were performed using GraphPad Prism (La Jolla, CA, United States) and Microsoft Excel analysis. Appropriate statistical tests (Student’s *t*-test, ANOVA) were determined after discussion with NCCU biostatistics faculty.

## Results

### *In vitro* and *in vivo* Vaping Models Display Acute Signs of Toxicity and Inflammation

Our previous studies have evaluated the differences in toxicity of resting/“unvaped” e-liquids upon human pulmonary cells (Zhang et al., 2020). Though our data demonstrated clear increased inflammatory responses and toxicity associated with the e-liquids, our more recent work attempts to evaluate e-liquids after the “vaping” process, that is, heating the e-liquid to aerosolize it into an inhalable vapor. We have also recently developed a method to “vape” e-liquids and also to approximate an appropriate “puff” rate during the process, leading to the production of an e-liquid distillate or “vaped” e-liquid.

**FIGURE 1:**
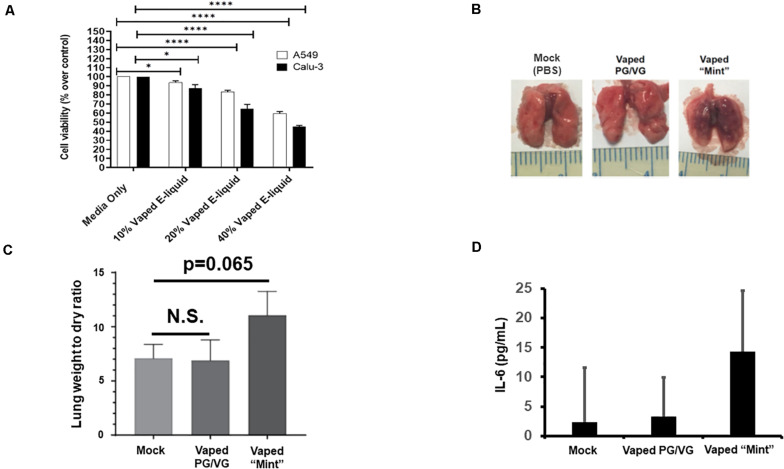
Acute *in vitro* and *in vivo* models of vaping indicate cytotoxicity and pro-inflammatory responses. **(A)** A549 (2.0 × 10^4^) and CALU-3 (3.0 × 10^4^) cells were plated overnight in 96-well plates. Vaped e-liquids were added at the indicated concentrations (vol/vol) in complete medium. The plates were then incubated for 24 h, stained for viability, and read using a PHERAstar plate reader. Media-only mock control treatments were also performed. *n* = 18–24 wells per pretreatment. **(B)** Mice [four mice (two males and two females)/ group] received either PBS (mock), vaped PG/VG vehicle, or vaped “Mint” e-liquid (10 μl, containing ∼20 μg of nicotine) once daily intranasal (IN) for 3 days and were then sacrificed on the 4th day. Representative macroscopic images of dissected lungs are displayed. **(C)** Lung wet/dry weight ratios of mice receiving either PBS (mock), vaped PG/VG vehicle, or vaped e-liquid. *n* = 4 mice (two males and two females) per treatment group. **(D)** The indicated whole lung supernatants were evaluated for IL-6 abundance via ELISA. ANOVA was performed to compare among different groups and compared with the mock or media only treatment using Dunnett’s *post hoc* test. Symbols and bars represent the mean ± SEM compared with the media only treatment (*P < 0.05, ****P < 0.001). N.S., not significant.

For our preliminary studies, we exposed A549 and CALU-3 human pulmonary epithelial cells to various concentrations of the vaped e-liquid, with toxicity assessed after 24 h (Figure 1A). Our results clearly delineate an increasing cytotoxicity with increasing concentrations (vol/vol) of the vaped e-liquid. We next evaluated the toxicity of our vaped e-liquid using our mouse model. To evaluate *in vivo* pathology, we delivered this same vaped e-liquid to mice [10 μl, containing ∼20 μg of nicotine, which is an equivalent amount of nicotine compared with the 10% cell treatments (Figure 1A), intranasal] intranasal (IN) for 3 days to evaluate acute pathology associated with vaping. On day 4, the mice were euthanized, and lungs were removed for analysis. Excised lungs from animals exposed to the vaped vehicle (PG/VG) visually appeared similar to the mock control, with no visible signs of inflammation or necrosis. However, upon examination, the lungs from the vaped e-liquid-exposed mice appeared both inflamed and darkened (Figure 1B). Lung weights were then measured to evaluate the “wet/dry” ratio (Parker and Townsley, 2004; Xu et al., 2006), which is a clinical measure of acute lung injury (Figure 1C). From the whole lung soluble lysate, we also observed an increase in the pro-inflammatory cytokine IL-6 in the mice exposed to the vaped e-liquid, while the vehicle-treated mice were more similar to the mock-treated animals (Figure 1D).

### Modeling a SARS-Like Infection in Vaping Primed Lungs

**FIGURE 2:**
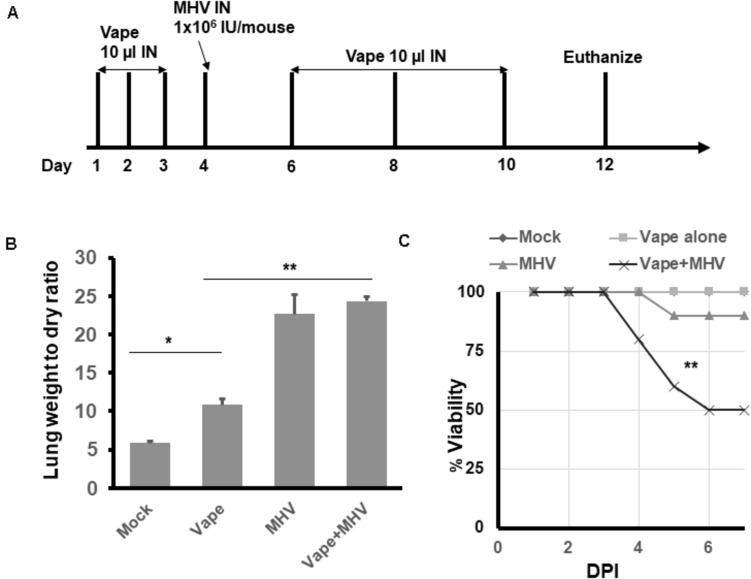
Vaping contributes to increased pulmonary pathology in mice infected with MHV-A59. **(A)** Study design. **(B)** Lung wet/dry ratios of mice receiving either PBS (mock), vaped e-liquid, infection with MHV or vaped and infected with MHV (compared with mock). All lungs were harvested on day 12. *n* = 4 mice (two males and two females) per treatment group. **(C)** Viability study of vaped mice or mice infected with either MHV or vaped + infected with MHV (compared with mock). *n* = 4 mice (two males and two females) per group. The mock and vape-alone groups overlay each other so that the mock is not visible. ANOVA was performed to compare among different groups and compared with the mock control using Dunnett’s *post hoc* test. Symbols and bars represent the mean ± SEM compared with the mock control (*P < 0.05, **P < 0.01). N.S., not significant.

To evaluate the effects of vaping on coronavirus pathogenesis, the murine coronavirus MHV-A59 was selected due to its published ability to induce SARS-like pneumonia in IN-exposed mice (Yang et al., 2014). Using our acute vape exposure model, we exposed animals to 1 × 10^6^ infectious units (IU) of MHV-A59 IN (a published sub-lethal dose) and monitored the mice for 8 days post-infection before euthanizing the animals (Figure 2A). We observed MHV-infected lung weights to be approximately five- and 2.5-fold higher than the mock and vape-alone treatment, respectively, indicating signs of clinical pneumonia associated with successful viral infection. While vaping appeared to exacerbate MHV-dependent pneumonia, this difference was not significant (Figure 2B). Viability of the mice was observed over time post-exposure to MHV (Figure 2C). While the vape-alone group suffered no mortality, the sub-lethal dose of MHV inoculum was confirmed by the limited mortality observed in the MHV-infected animals (MHV alone). However, mortality was significantly increased in animals exposed to the vaped e-liquid and MHV infection.

These results suggest that MHV infection leads to pulmonary pneumonia within our model and that the contribution of vaped e-liquid exposure to MHV-dependent pulmonary pathogenesis is a decreased survival rate for the co-exposed animals (Figure 2C).

### A Role for Ca^2+^ Flux in MHV-A59-Infected and Vape-Primed Lungs?

Our previous work (Ghosh et al., 2020; Zhang et al., 2020) as well as the work of others (Rowell et al., 2020) have implicated the potential role of increased intracellular Ca^2+^ as the mechanism of e-liquid-induced cytotoxicity *in vitro* (Figure 1A). In particular, it has been questioned if e-liquids may activate endoplasmic reticulum (ER)-resident Ca^2+^ release as the mechanism of toxicity (Ghosh et al., 2020). To further examine this premise, we utilized our model and the well-described inositol triphosphate (IP_3_) receptor antagonist 2-APB, i.e., the receptor that regulates an ER-resident calcium channel that releases calcium into the cytosol when activated (Maruyama et al., 1997).

**FIGURE 3:**
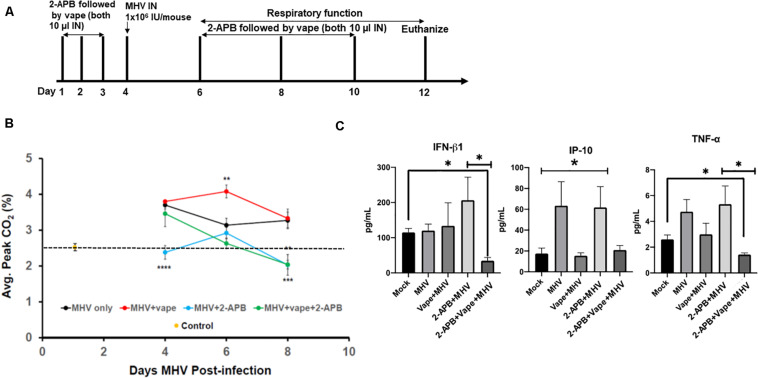
2-APB treatment reverses some of the effects of MHV infection in mouse lungs. **(A)** Study design. **(B)** Average peak CO_2_ levels detected from the treatment groups 4, 6, and 8 days PI. The data were acquired using a SomnoSuite low-flow anesthesia system equipped with a nose cone. Mice were sedated with ketamine and monitored for ∼2–3 min to acquire peak CO_2_ wave forms. *N* = 6 mice (three males and three females) per group. The dotted line is a reference point for the control. **(C)** Inflammatory cytokine analysis of BAL fluid from mice infected with MHV, vape + MHV, 2-APB + MHV, and 2-APB + vape + MHV (compared with mock control). ANOVA was performed for multigroup comparisons (Tukey’s multiple comparison tests). The statistically significant IP-10 comparisons (*P < 0.05) are made between the mock and MHV groups, and the mock and 2-APB + MHV groups only. Symbols and bars represent the mean ± SEM compared with the mock control (*P < 0.05, **P < 0.01, ***P < 0.005, ****P < 0.001). N.S., not significant.

Our acute vaping model followed by MHV infection on day 4 was performed in the presence and absence of 2-APB IN (10 μl of a 500-μM stock, which is 44 ng/g body weight) on each day the animals received the vape treatment, that is, days 1–3 prior to infection and days 2, 4, and 6 post-MHV infection (Figure 3A). However, in each case of dosing, the 2-APB was provided 30 min before the IN vaped e-liquid was dosed, that is, a prophylactic treatment. Our data indicate that the animals receiving the 2-APB treatment displayed improved respiratory function, as measured via spirometry (peak CO_2_ output, SomnoSuite analysis), compared with either the MHV- or MHV + vape-treated groups, with the MHV + vape-treated group displaying the worst overall respiratory function (as seen on D6 of the time course, Figure 3B). Interestingly, 2-APB treatment also ameliorated respiratory function within MHV-alone groups, indicating an important role for Ca^2+^ signaling in viral pathogenesis in general. In addition, and in contrast to the treatment groups illustrated within Figure 2C, there was no mortality observed within the MHV + 2-APB- or MHV + vape + 2-APB-treated groups, i.e., 100% viability for these two groups was observed across the entirety of the treatment regime (data not shown).

Inflammatory cytokines were then assessed from bronchoalveolar lavage (BAL) fluid. MHV infection led to increases in pro-inflammatory cytokines, while the effect of the 2-APB treatment proved confounding and difficult to interpret (Figure 3C). For example, the 2-APB treatment did appear to increase the INF-β1 level when provided together with MHV infection though not significantly – a result we cannot currently explain. Our cytokine profiles from our preliminary analysis do appear to demonstrate the activation of TNF-α in the MHV-infected lungs, which is diminished to near mock-treated animal levels in the presence of the 2-APB treatment but only with the dual MHV + vape treatment. Again, the 2-APB + MHV treatment group has activated cytokine levels. In contrast, the IP-10 level is not increased in the MHV + vape group. Therefore, the 2-APB + vape + MHV treatment does not appear to alter the abundance relative to the mock control.

**FIGURE 4:**
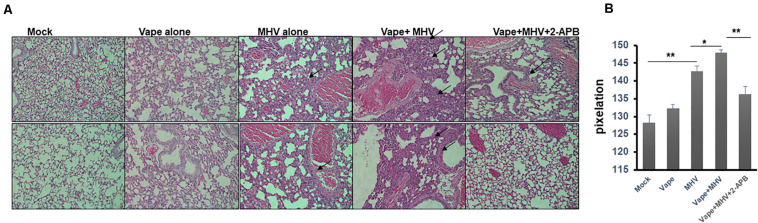
Histopathology indicates that 2-APB treatment reverses some of the effects of MHV infection in mouse lungs. **(A)** Histopathology of lung tissues. H&E staining of sections of lung tissue isolated from mock-, vape alone-, MHV alone-, vape + MHV-, and vape + MHV + 2-APB-treated mice. Alveolar wall thickening and the infiltration of inflammatory cells into the interstitial spaces were particularly observable in the lungs from the MHV-alone and vape + MHV mice (indicated by arrows). However, these features were much less pronounced in the vape + MHV + 2-APB animals. 200× magnification. **(B)** Pixelation was quantified as a measure of inflammatory foci with five images per lung. Student’s *t*-test was performed between groups. Symbols and bars represent the mean ± SEM compared with the mock control (*P < 0.05, **P < 0.01). N.S., not significant.

Importantly, the H&E lung histology of the MHV + vape + 2-APB-treated group was more similar to the control animals than to the MHV- or MHV + vape-treated groups (Figures 4A,B). Constriction of air space and consolidation of alveoli (demonstrative of viral pneumonia) were both demonstrated in MHV and vape + MHV lungs, while inflammation was diminished in the presence of 2-APB treatment.

In sum, these data suggest that vaping may significantly exacerbate the severity of pulmonary coronavirus infection, leading to increased pulmonary infiltrate of inflammatory cells (Figures 4A,B). Importantly, this disease burden may be mediated by Ca^2+^ signaling, such that a calcium antagonist may alleviate pathology.

## Discussion

In the latter part of 2020, the COVID-19 pandemic brought to light a glaring lack of knowledge of the causative factors that may contribute to the severity of acute viral pneumonia due to SARS-CoV-2. SARS-CoV-2 has rapidly spread across the globe and affected a diverse populace, leading to a high variability in disease prognosis. Health professionals and infectious disease experts are still unclear as to which risk factors may contribute to disease severity. Tobacco smoking is known as a major risk factor for the development of disease and disability. Despite package health warnings, advertising bans, and increased taxes on tobacco products, an estimated 20% (1.3 billion people) of the world’s population (36.5 million people in the United States) still smoke tobacco (Rom et al., 2013; Jamal et al., 2016). The role of vaping in pulmonary disease initiation and progression is still relatively unknown.

Herein, we have developed a mouse model to evaluate vaping/E-Cig exposure as a risk factor for coronavirus-dependent pulmonary disease. While exposing mice to vaped e-liquid IN, we observed increasing, though not significant, levels of acute inflammation (Figure 1), demonstrating pathology. Using this model, we went on to evaluate the effects of vaping upon coronavirus-dependent pulmonary pathogenesis using a mouse-tropic coronavirus strain, MHV-A59 (Figure 2). As we hypothesized, our preliminary studies indicate that vaped e-liquid increases the mortality and the pathology of MHV-induced pulmonary infection. However, vaping appears to dysregulate cytokine activation in our studies (Figure 3C), suggesting a complex and complicated role for vaping-related Ca^2+^ mobilization in inflammation and perhaps ultimately in respiratory disease development. This observation is pertinent and topical as E-Cig use (particularly among minors and young adults) rates are increasing (King et al., 2018), potentially indicating this population is at a greater risk for hospitalization due to coronavirus infection.

However, we must also acknowledge the limitations of our study. We employed *in vitro* immortalized pulmonary epithelial cells for our preliminary studies. As such, we have not yet performed similar studies with primary cells, which would provide more biological significance as the data obtained from immortalized cell lines do not always accurately replicate the data obtained when using primary cells (Kaur and Dufour, 2012). Such studies, which will also include using air–liquid interface (human airway epithelial cells) culture systems, will be incorporated into further works to evaluate the effects of vaping and MHV upon ciliated epithelial cell function as well as better model potential drug effects. Next, as indicated in Figure 1, our data indicate increasing trends for both lung weight/dry ratio and IL-6 level due to IN exposure to the vaped e-liquid. Even so, the data are not statistically significant, likely owing to the small sample size or “n” utilized within the context of this small preliminary study. Thus, statistical differences might be reached if using a larger sample size. Next, we have employed an intranasal route of exposure using a vaped e-liquid distillate, which does not exactly recapitulate the vaping experience. Our use of a condensed vaped e-liquid distillate has been termed an “intermediate approach” but is not a direct exposure route. In addition, the use of the e-liquid distillate does overcome some of the shortcomings of direct exposure. For example, a weakness of direct exposure routes is that E-Cig topographies, also known simply as smoking behavior and including such characteristics as puff duration, are poorly understood and will change as new E-Cig devices emerge (DeVito and Krishnan-Sarin, 2018). This fact is in contrast to traditional combustible cigarette puff topographies, which are well studied and defined. Second, doses received during direct exposures can be variable and include such issues as dermal and oral absorption, i.e., mice licking deposited vape aerosols off of their fur when whole body exposure is performed, among others (Oyabu et al., 2016; Noel et al., 2018). However, there is clearly value to direct exposure routes such as whole body and nose-only exposure routes, and we are working to develop these models, in particular, the nose-only exposure model, and to compare/contrast these direct exposure data with our own (Miyashita et al., 2018; Gotts et al., 2019).

Our previous work also focused on the role of Ca^2+^ signaling in E-Cig-related cytotoxicity *in vitro* (Zhang et al., 2020). Therefore, we evaluated whether an antagonist for calcium signaling (2-APB) could alter the prognosis of the animals within our treatment groups. Indeed, our results do suggest that pathology is diminished in the 2-APB-treated mice [both the spirometry and gross pulmonary histology (Figures 3, 4)], suggesting potential novel therapeutic interventions that may currently exist and that can be improved and repurposed. For example, based upon the understood mechanism of 2-APB, which is to perturb the ER stress pathway by inhibiting ER-resident Ca^2+^ release into the cytosol (Maruyama et al., 1997), 2-APB could potentially prevent viral replication (Tanaka et al., 2013; Jiang et al., 2020). Again, the potential pro-inflammatory role of 2-APB will require a more comprehensive study design that includes more subjects and variable times, doses, and types of exposures to confirm our results in future studies. However, it is possible that the normal lung response to MHV is to increase IP-10 and TNF-α levels, although these data were not significant in our study. Therefore, with the dual exposure, i.e., adding the vape treatment, this antiviral response is prevented. This lack of a response may be more apparent in the animals who died, which is an avenue of study for our future directions.

In sum, our model suggests that vaping exacerbates coronavirus-dependent pulmonary disease in mice. However, the exact mechanism of disease in MHV-infected and E-Cig condensate-treated mice remains to be established, which will benefit from our future studies that will include larger cohorts and a more robust and rigorous experimental design. Therefore, this model has a potential use for testing promising therapeutic interventions.

## Data Availability Statement

The original contributions presented in the study are included in the article/supplementary material, further inquiries can be directed to the corresponding author.

## Ethics Statement

The animal study was reviewed and approved by the North Carolina Central University IACUC.

## Author Contributions

VS and RO were responsible for the study concept and design, acquiring the data, data analysis, manuscript writing, manuscript editing, and study supervision. De’JP, RZ, and MJ were responsible for acquiring the data, data analysis, and manuscript editing. All authors contributed to the article and approved the submitted version.

## Conflict of Interest

The authors declare that the research was conducted in the absence of any commercial or financial relationships that could be construed as a potential conflict of interest.

## Footnotes

1 juul.com
